# Analysis of the Article "Cas Rares," in the Dict. des Sciences Medicales

**Published:** 1823-09-01

**Authors:** 


### *Analysis of the Article* "Cas Rares," *in the Dict. des Sciences Medicales.*

"Apparent rari nantes in gurgite vasto." Virg.

Considering how small a proportion of our Medical Readers can peruse, or have access to, the colossal work abovementioned, we trust that in condensing a very curious Article entitled "Extraordinary Cases" by M. Fournier, we shall merit the approbation of a great majority of the friends of this publication.

M. Fournier declares, that he has made a very scrupulous selection of facts for this article, rejecting all histories of cases that are not attested by unquestionable authorities. He divides the "cas rares" into three classes or sections, as they affect the anatomical structure—the physiology or functions—and, lastly, the laws of the animal economy.

FIRST SECTION.

M. Fournier remarks, that of all organs or parts of the body, the brain is found to be least frequently in a state of aberration from natural structure, independent of disease. The relative *size* of this organ, therefore, occupies the first pages of the article. Dr. Louis Valentin has published the description of a cranium, very remarkable for its dimensions, which is preserved in the Cabinet of Natural History at Marseilles. The man's name was BORGHINI, who died in the abovementioned city in 1616, at the age of 50 years. He was but four feet in height, yet his head measured *three feet* in circumference. The bones were thin, and there was an opening, the size of a crown-piece, where the sagittal meets the coronal suture. Dr. Valentin facetiously remarks, that although poor Borghini had so much *brain*, he had very little *wit*;—so little, that it became proverbial at Marseilles to say to any stupid fellow—"You have no more wit than Borghini!" When he became advanced in years, he was obliged to have a cushion on each shoulder to support his head.

2. The next "cas rare" is a Moor at Tunis, whose head was of such a prodigious size, that people gazed at him wherever he went. His nose was four inches long, and his mouth was so capacious, that he could bite through a melon with the rind on, as easy as another person could bite through an apple! This man was also imbecile.

3. The third was *Philip Sormet*, a native also of Marseilles, and who died there in 1807, aged 71 years. He had a prodigious head. He never lay down in a bed for thirty years before his death, nor ate more than one meal a day. He was not, however, quite so stupid as his townsman Borghini, for he was a great compiler, and also a *critic*. His voluminous manuscripts were found, after his death, to be entirely extracts from authors, (his criticisms were always verbal,) which he had been compiling, or rather *piling*, from every book which came in his way. He used to pass by Borghini's head every day in the public library, but affected to take no notice of it. He would there collect around him forty, fifty, or sixty volumes, and often go to sleep among them with the pen in his hand.

4. The fourth was a young man at Lucca, whose head was more than *three feet* in circumference, and face fifteen inches long! The rest of his body was well proportioned, and his intellectual faculties remarkably bright. He died of apoplexy at the age of thirty.

5. Passing by several not very interesting cases of aberrations from Nature, in the hair and eyes, we have the description of a curious Cyclopean monster by Eller. In 1755, a child was born with an enormously large head and frightful visage. In the middle of an ample forehead was an only eye, large but without eye-brows or eye-lids, and above the eye was a thick cylindrical excrescence, resembling a penis, with urethral canal, prepuce, and glans!

6. There is nothing very worthy of notice among the cases of peculiarity of structure in the ears, nose, and mouth. Two curious instances of very long tongues are brought forward, and they were in *females*. M. Fournier saw a woman at Berlin, whose tongue was amazingly large, but thin as a cat's. When this woman laughed, her tongue covered the whole of her mouth, and hung out like folds of drapery. It was always cold, and communicated a most frigorific sensation to the hand of another person. The same author knew a handsome young woman, fifteen or sixteen years of age, who, although she had a long neck, could extend her tongue to her breast without stooping her head!

7. A third case is related of a young Portuguese lady, who, instead of a tongue, had only a small eminence resembling a nipple. This eminence had a slight contractile and expansive motion, and yet this young lady contrived to *speak* distinctly, though she was forced to use her finger in pushing the chewed aliment back towards the pharynx to be swallowed!

8, 9, 10. Morgagni mentions a man in whom the epiglottis was wanting, though he spoke and swallowed without difficulty. Vicq. D'Azir relates a case of a man in whom there was a dilatation of the œsophagus resembling the crop of birds. In swallowing, the food passed into the sac, and there remained till he vomited it up; this man died exhausted. A young woman had a difficulty of swallowing from her infancy. Towards the period of menstruation, it got worse, and the disease increased rapidly, and was always aggravated by exercise. At length deglutition became impossible, and the poor young woman died. On dissection, the cause of the malady was found to be an aberration in the course of the subclavian artery, which passed *between* the trachea and œsophagus, compressing the latter, and preventing deglutition.

11, 12. Diemerbroeck dissected a subject in which the diaphragm and mediastinum were entirely wanting; and where the lungs formed but one lobe. M. Fournier himself examined the body of a soldier, who had been killed in a duel, and in whom the heart occupied the right side of the thorax, and the lungs (in one lobe) the left. The stomach and intestines were naturally situated, but the liver was placed in the left side. Telasius asserts, that he dissected a Roman soldier, in whom there was not a vestige of *heart* to be found! However *heartless* a set the degenerated inhabitants of the "eternal city" may now be, we think M. Fournier might have placed this among the "cas rares *fabuleuses.*"

13. Passing over a long list of females who had three, four, and even five breasts, we come to a curious malformation in the body of an officer who was opened and where the whole of the stomach and part of the colon were found in the left cavity of the thorax, into which they had passed through cartaliginous rings in the diaphragm that adhered to these viscera as they entered. The colon, after entering the thorax through the left side of the diaphragm, returned through an aperture in the middle of that muscle, and then pursued its usual route. The right cavity of the chest was filled with water, (the lung being wasted) and the heart was much enlarged.

14. "An *adult*, who died of dropsy, was opened. The liver and spleen were entirely wanting. The vena portæ opened immediately into the inferior cava. We have nothing analogous to this case, says Fournier, upon record, but the authority of *Lieutaud* prevents us from rejecting it as suspicious."—Granting that this was the case, it does not detract from the importance of the *hepatic* functions, in the animal œconomy, any more than the case of the Roman soldier proves the *heart* to be an unnecessary organ in the human frame.

15. The following case is so remarkable, that we shall translate it verbatim.

"*A girl,* 14 *years of age, well grown, and of a beautiful figure, had neither genital organs nor anus, nor any vestige of them, the parts where these are usually seated being covered with natural skin. This young person ate with an appetite; slept well; and enjoyed excellent health. Every two or three days she felt, in the umbilical region, a deep seated pain accompanied by an increasing uneasiness, till at length she vomited fæcal matter. From that moment the pain and uneasiness would cease, and after washing the mouth carefully, and chewing some aromatic substance, the disagreeable smell of the excrements would subside.* The urine was evacuated from her breasts. *("Les urines s'evacuaient par les mamelles.") It would have been desirable to observe this girl at the epoque of the commencement of the catamenial period, to see by what means Nature would deliver herself from the menstrual fluid, or what accidents would happen from its retention in the uterus.*"

16. M. Fournier relates the case of a woman, 22 years of age, whom he found to have the anus imperforate from birth. The rectum opened without any sphincter into the vagina.

17. Zacutus Lusitanus relates the case of a child who was born with a membrane over the anus, and passed the excrements, per urethram, for three months, when the natural passage was established, and the fæces gradually resumed their proper course. Several other well attested cases of the fæces passing, even for years, through the urethra, are related, but the above specimen is sufficient.

18. A man was dissected by Lieutaud, in whom the urinary bladder was wanting. The ureters were as large as small intestines, and terminated at the lower part of the pelvis, in the urethra. He died at thirty-five.

19. A man, aged 32, passed his water by the navel. It came out with a jet, as though a sphincter existed there.

20. Lieutaud relates a case, (the only one on record,) where no vestige of a womb was discovered on the dissection of a woman. *Coitus erat difficilis.*

21. Professor Lobstein, of Strasburg, dissected a woman who had two distinct wombs. He thinks that where there is a superfœtation or double conception, there must be two wombs, as above.

22. A single woman of *fourscore* years of age, ("Une fille de quatre-vingts ans,") had the hymen nearly circular, with only a small perforation in the middle! It is preserved (as one of the greatest curiosities in all *France)* by M. Sue, Member of the Academy of Surgery. M. Fournier says, this is a most surprising phenomenon, since the hymen is so early destroyed, in general, by the most *innocent introductions.* "Puisque l'hymen se détruit de fort bonne heure, par les *introductions les plus innocentes.*"

23. Vallesnieri dissected a female in whom were found two uteri; the orifice of one opened into the vagina, of the other into the rectum. M. Fournier says, there is no doubt but that *coitus per anum* would have been followed by conception. He grounds this opinion on the curious case related by the celebrated Louis, of a young lady who had a congenital imperforation of the external organs of generation. This girl menstruated per anum. She was solicited in marriage by a young man to whom she was attached. After much resistance she confessed to him the secret.—"Dans le delire de sa passion, il supplia son amante de consentir a ce qu'il s'unit a elle par la seule voie qui fut practicable; elle souscrivit a tout et bientot elle devint mere. L'accouchment a terme, d'un enfant bien formé, eut lieu par l'anus." Louis made this the subject of a thesis, and was prosecuted by the parliament of Paris; and the doctors of the Sorbonne *interdicted* him for addressing to the casuists the following question:—"In uxore, sic disposita, uti fas sit, vel non? judicent theologi morales." The Pope, however, more philosophic than the Parliament or the Sorbonne, gave absolution to Mons. Louis, and his Thesis was published in 1754.

From the Cases of Hermaphrodites we shall not select any, since M. Fournier is of the same opinion with the physiologists on this side the water, that they are all merely *imperfect* males or females, chiefly the latter, and never conjoining the two sexes in one.

At the end of hermaphroditism, it is asserted, on the authority of Hufeland, that the body of a child, three years of age, was lately opened at Berlin, in which there was not the slightest trace, either internally or externally, of any part of the genital organs peculiar to either sex.

We have next a dissertation on those causes which change the colour of the human skin, and on *nevi materni*. M. Fournier believes, contrary to Richerand, and most of the physiologists of the present day, that strong impressions on the mother, during pregnancy, will indubitably occasion nevi materni, though the vulgar have exaggerated the circumstance by attributing every trifling mark to causes of this kind. His remarks on Albinos have nothing novel in them.

M. Fournier concludes the first section of this article with the description of a man, who lately died at Paris, and was examined by the *Ecole de Medecine*, from whose procés-verbal the details are taken. Marc Catozze, commonly called the little dwarf, was born at Venice, of robust parents, and had several brothers, strong and well formed. He died a few years ago at Paris, aged sixty-two years. The trunk of the body exhibited nothing remarkable, and appeared to belong to a person of about five feet six inches in height; there was, however, no scrotum. The upper extremities consisted of merely two prominent shoulders, to which were attached two hands, without either arms or fore-arms. The lower extremities consisted of a flattened plane, (une fesse applatie) to which were appended two ill-formed feet, without thighs or legs. As Catozze could not feed himself, or bring his hands to his mouth, Nature had furnished him with a curious under jaw, which he used as an elephant uses his proboscis. At least, with this he managed his victuals very well. Catozze was very fond of women, wine, and good cheer. He was also fond of society, and spoke and wrote several languages. On dissection, several peculiarities were observed in the interior of the trunk, but they need not be recorded here.

### Section. II.

*Or, singular aberrations from the natural functions and actions of life. This Section is alphabetical. It commences with abstinence.*

1. On the authority of Vanderviel, a madman, who conceived himself the Messiah, totally abstained from food and drink during a space of *seventy-one days!* All that he did was to smoke and wash his mouth. His health was not impaired. He passed no excrement in that period. Credat judeus!

2. In the Memoirs of the Academy of Sciences for the year 1761, another case of abstinence is related, which lasted *four* years, accompanied with very curious circumstances! Scepticism and credulity are, they say, frequently united!

3. Extraordinary lactation.—In the year 1810, a poor woman, of the name of Charles, was delivered of male twins; but being of a weakly constitution, and unable to suckle them both, she applied one of them to the breast of his *grand-mother*, aged 65 years, and in the twenty-ninth year of her widowhood. It was "great *cry* and little wool" with the poor boy for the first few days, but afterwards the milk came kindly and copiously for twenty-two months, and this boy became the stronger of the two. This would have been encouragement to Johanna Southcote, as, in all probability, young Shiloa would not have required a wet nurse, but have drunk from the lactiferous fountains of his holy mamma. The above is on the authority of Dr. Montegre, who has published the circumstance in the Gazette de Santé.

4. A case of catalepsy is related by M. Fournier himself, and which was caused by the suppression of an habitual diarrhœa. This diarrhœa had continued five years, (having succeeded the cessation of the menses,) during which time she enjoyed excellent health otherwise; wishing, however, to get clear of the diarrhœa, she applied to a Charlatan, who gave her strong astringents, which suppressed the intestinal flux, and instantly brought on a cataleptic fit. M. Fournier was called in, and by purgatives, &c. restored the accustomed evacuation, and removed the catalepsy,

5. The following extraordinary effect of musk on the penis is absolutely incredible, though related by Borelli. "Borelli dit avoir connu un homme qui se frotta le membre virile de musc avant le coit; il l'exerça et resta *uni a sa femme comme les chiens le sont avec leur femelles.*" (How can this be possible?) "Il fallut lui donner une grande quantité de lavemens, afin de ramollir les parties et obtenir la separation des deux individus."

6. Cabrol relates the curious, but dreadful effects of cantharides, taken as an aphrodisiac. A man swallowed *two drachms* of this active medicine, and afterwards effected *eighty-seven coitus in una nocte!* (L'insensé approcha sa femme quatre-vingt-sept fois pendant la nuit,) besides several seminal emissions. Death was the consequence.

7. Several cases of spontaneous human combustion are next related. M. Fournier observes at the close, that the fact of spontaneous combustion is now so indubitably proved, that scepticism on the subject would be unreasonable.

8. After the details of an extra-uterine conception, a case is related, where a fœtus was found in the abdomen of a child. A similar case is related in one of the volumes of the Medico-Chirurgical Transactions. A girl of 14 years of age, died of a pulmonary consumption; but had had from infancy a painful swelling in the left side. On dissection, a fœtus, imperfect in some of its parts, was found in a cyst attached to, and communicating with, the transverse arch of the colon. The French physiologists conceive, that in these cases, the containing and contained animals are congenital—viz. brothers or sisters.

9. Passing by several cases of extra-uterine conception, and among others, one where the fœtus, in two or three successive abortions, was brought forth by the mouth, we come to a curious case of constipation, where the man who was so affected, had fæcal evacuations every Wednesday only, and that from his infancy. He suffered no inconvenience. The other excretions were rather more copious than usual; the fæces were well formed, and in considerable quantity when passed on the day above-mentioned.

10. A gentleman is now living in Paris, in the 50th year of his age, whose intellectual faculties are of the first order, and who is intimately conversant in every species of literature. His style is so elegant that he is the delight of his readers, while his critical acumen is dreaded by every mediocre author, whose works come under his sensorial lash. His person is tall and meagre, his complexion pale and bilious. He may be said to sleep none—at least, he does not sleep more than a quarter of an hour in the course of the night. When he sleeps four or five hours, it is the certain precursor of a fit of sickness, which never fails to assail him in the course of twenty-four hours after this unusual somnolescence. He never passes a stool oftener than once in twenty-five or thirty days, and that by the aid of glysters. Purgatives have no effect on the stomach or bowels; his excrements are like little stones, in the form of sheep or deer's dung. He has no appetite, and eats very little. When ill, he will not eat any thing for a month together, living entirely on drink. He takes great exercise, walking sometimes above an hundred leagues, almost without resting; and it is at these times that he enjoys the best health. He is highly irascible on the least contradiction, but the goodness of his heart is never altered.

11. One of M. Fournier's most intimate friends, arrived at the age of 48 years, without drinking any liquid. He ate however, voraciously. He was never of course thirsty. For some indisposition he was twice bled, immediately after which he was seized with *hydrothorax*, and died.

12. Several instances are related of *rumination* in the human subject, similar to the rumination of animals; but they are unworthy of notice.

13. *Sweating of Blood.*—A woman, 46 years of age, had been subject to periodical hæmatemesis for a considerable time, for which strong astringents were imprudently administered, when the hæmatemesis suddenly disappeared, and was succeeded by diapédèse, or sweating of blood, which proceeded from all parts of the body in succession. It became periodical like the hæmatemesis.

14. Under the head of particular idiosyncrasies, the hæmorrhagic disposition of a female, named Smyth, in America, and her descendants of the male line, is related; but as this idiosyncrasy has been noticed in our own journals, it need not be repeated here.

15. Among the instances of longevity, Parr is not mentioned, but Henry Jenkins is said to have lived to the age of 189 years, and to have died in 1690.

16. M. Fournier relates the case of a *man* who *menstruated* regularly per urethram.

17. The following case, attested by the principal physicians and surgeons of Brest, must cast in the shade that of the *knife eater*, whose dissection excited so much curiosity, a few years ago, in this country.

A galley slave died at the Naval Hospital of Brest, of a complaint in his stomach, attended with cough and colicky pains. On opening him, the stomach was seen occupying the left hypochondrium, the lumbar and iliac regions of the same side, and stretching down into the pelvis. It was of a long square form, and contained the following substances; viz. a piece of a stave *nineteen inches long*, and half an inch in diameter; a piece of a broom-stick six inches long, and half an inch in diameter; another piece of the same eight inches long; ditto six inches long; twenty-two other pieces of wood, of three, four, and five inches in length; a wooden spoon five inches long; the pipe of an iron funnel, three inches long and one in diameter; another piece of funnel, two inches and a half long; a pewter spoon entire, seven inches long; another three inches long; another two inches and a half long; a square piece of iron weighing nearly two ounces. Various other articles, among which were nails, buckles, horns, knives, &c. the whole weighing about twenty-four English ounces. This poor creature was deranged in his intellects—a great glutton; and when he could not procure victuals to satisfy his voracity, he swallowed indigestible substances, as above, to lull the painful sensations of hunger. This case is attested beyond all doubt.

18. Passing by the famous Bijoux, who lived at the Royal Menagerie, and amused himself in classing animals by the forms of their excrements, we come to a singular personage, well known in Paris, where he died a few years ago, and was named *Tarrare*. This man's voracity would stagger all belief, were not the truth of the circumstances guaranteed by the most unquestionable testimonies, among which it is only necessary to mention Professor Percy. At 17 years of age Tarrare weighed only one hundred pounds, and yet he could devour, in the space of twenty-four hours, a quarter of beef as heavy as his body! At the commencement of the revolutionary war he entered the army, but here he was so scantily supplied with food, that he soon got ill, and was conducted to the military hospital at Soultz. On the day of his entrance he got four rations, which only serving to whet his appetite, he devoured every kind of refuse victuals in the ward, then searched the kitchen, dispensary, &c. devouring every thing, even the poultices, that came in his way! In the presence of the chief physician of the army, Doctor Lorenze, he ate a live cat in a few seconds, leaving nothing but the larger bones! In a few minutes, he devoured a dinner prepared for fifteen German labourers, and composed of various substantial dishes. After this *tiffin*, his belly appeared like a small balloon! As the French in those days turned every thing to account, the commander in chief had him brought before him, and after treating him with thirty pounds of liver and lights, he caused him to swallow a small wooden case, in which was enclosed a letter to a French officer, then in the hands of the enemy. Tarrare sat off, was taken prisoner, beaten and confined. He passed by stool the case with the letter, before he could see the officer, but immediately swallowed it again, to prevent its falling into the hands of the enemy. In another hospital where he was confined, the nurses frequently detected him drinking the blood which had been drawn from the sick; and when all other sources failed, he repaired to the dead-house, and satisfied his frightful appetite on human flesh. At length a child of fourteen months old disappeared all at once, and suspicions falling on Tarrare, he also disappeared for four years, when he was recognized again in the civil hospital of Versailles, where he ended his miserable career. Tarrare's voracity far exceeded that of the French prisoner taken in the Hoche last war, and whose gluttony is attested by the late Dr. Johnstone. Indeed Tarrare seems to have realized the fable of *Erisichthon*, who, according to the poet, devoured what would have supported a whole town—a whole people:

——— "quod urbibus esse,
Quodque satis poterat populo."—Ovid. Met. Fab. 18.

19. Two instances of precocious puberty are next related. The male instance is not more remarkable than the one lately published in the Medico-Chirurgical Transactions, and therefore need not detain us. The other is that of a girl, six years of age, who menstruated regularly. The remainder of this section contains nothing worthy of note.

### Section III.

*Or, curious Cases of Disease.*

1. An ophthalmy, accompanied by ulcerations of the cornea, was cured by extracting some carious teeth of the same side. This hint is worth attending to, since it is pretty certain that many diseases are kept up, or even caused, by sympathy with other parts in a state of disease.

2. Dr. Forlenze found an ossification of the eye in a subject at Paris. The sclerotic was natural; but the iris, the cyrstalline lens, and the vitreous humour, were completely ossified. The same gentleman operated for the cataract in presence of M. Fournier, where the crystalline lens was perfectly ossified.

3. M. Imbert removed from the face of a Mons. Perrier de Gurat, a remarkable sarcomatous tumour affecting the nose, which was divided into five lobes, covering the greater part of the face, and rendering the visage so hideous, that the poor gentleman was obliged to seclude himself from society. It weighed two pounds. It was removed with safety.

4. A stone, the size of a pigeon's egg, was removed from under the tongue of a man 37 years of age. It had caused the most exquisite pain and profuse salivation.

5. Saxonia relates the case of a woman, who for a long time suffered the most excruciating pain on the *right* side of her head. On dissection, the *left* side of the brain was suppurated, while the right was perfectly sound.

6. A young lady died of long-continued and severe epileptic fits. On dissection, ten or twelve bony productions, half an inch in length and very sharp pointed, stood out from the cranium near the superior longitudinal sinus, penetrated the dura mater, and injured the brain.

7. M. Fournier relates the following case himself:—

"*An unmarried woman,* 48 *years of age, was suddenly seized with*

*a pungent and lancinating pain in every part of the head, except the occipital region. This pain was excruciating, the patient appearing phrenitic, and uttering the most lamentable shrieks. The paroxysms of pain recurred every five or six minutes, and lasted about a minute each time. The patient was pale, and the pulse slow and weak during the intervals; but as the paroxysm came on, the countenance reddened, and the pulse became frequent and bounding. M. Fournier learnt from the patient, that seven or eight months before this she had received a blow on the head by the key of a door, which had caused sharp pain for some time, followed by vertigo, which went off without any medicine. M. Fournier attributing the present symptoms to the former injury, prescribed a grain of the tartrite of antimony, which vomited her once. The second day, two grains were prescribed, and she vomited several times; the third and fourth days, the same medicine and the same result. The fifth day, three grains; the sixth, four grains of the tartrite, when violent vomiting and straining succeeded, during which there burst, all at once, a quantity of inconceivably fetid pus from the mouth, nose, and ears. From this moment the head-ache vanished, as if by magic. Three days afterwards, an emetic brought away a small quantity by the ear; after which the patient soon recovered completely.*"

This is certainly a curious case, and a curious method of treatment. If M. Fournier suspected inflammation or suppuration within the cranium, a repetition of emetics seemed a singularly hazardous practice. We should be inclined to think that it was abscess in the mastoid cells, and that the matter had made its exit partly by the Eustachian tube or tubes, and partly by the external ear, having burst the membrana tympani.

8. A man received a wound by a musket bullet, which penetrated into the brain, through the frontal sinus. He recovered, in a short time, without any untoward accident, and enjoyed excellent health for four months. At the end of this period he was suddenly seized with a lethargy, and died convulsed. The ball was found in the medullary substance of the brain, half an inch above the anterior portion of the left lateral ventricle.

9. A bullet remained in the substance of the brain during a period of two years, and finally proved fatal. Another man lived fourteen years, during which time a piece of iron as large as a finger was lodged in the brain, and eventually was dislodged by the efforts of Nature.

10. Blegny relates the case of a lady who was long afflicted with severe pain in the head, accompanied by fever. At length, she lost her sight; and her sufferings were so great that she died. On examination, a stone, the size of a bean, was found at the origin of the optic nerve, in the substance of the thalamus.

11. Guersent relates an interesting case of rupture of the œsophagus, in a girl seven years of age, by vomiting. The symptoms were, fever, thirst, sighing, nausea, convulsions; the tongue thrust out of the mouth, the skin of a crimson colour. Extreme debility followed the convulsions; the face became of a violet colour, the pupils dilated, the skin burning hot, deglutition painful, respiration difficult; death, in thirty-six hours. An oval oblong rupture of the œsophagus, a little before that tube penetrates the diaphragm, was the cause of all. The right thoracic cavity was filled with a brown liquor. A stilette passed from the opening in the gullet into the stomach.

12. A man, 60 years of age, swallowed a piece of meat, which stuck in the œsophagus, near the lower end of the tube. Alarming symptoms followed. The surgeon could not displace the foreign body. He opened the median vein of the right arm, and injected four grains of emetic tartar dissolved in an ounce of water. About a minute afterwards, the patient vomited up the piece of meat.

13. Dr. Latour, first physician to the Grand Duke of Berg, relates the following very interesting case. A soldier received a musket shot in the breast, and was carried off the field of battle almost dead. A tremendous hæmorrhage ensued, but it had nearly ceased by the third day. After this, the strength of the patient gradually recruited, and suppuration succeeded the hæmorrhage. Several pieces of the fractured rib now came away; at the end of three months the wound was cicatrized, and the man's health was re-established, with the exception of palpitation of the heart, which annoyed him for three years afterwards. Six years after the accident, he died of a disease unconnected with the wound. M. Mausion, chief surgeon of the hospital at Orleans, opened the body. The cicatrix was deep and extensive. Pushing his researches farther, he discovered the ball encased or lodging (enchatonné) in the right ventricle of the heart, covered over, in a great measure, by the pericardium, and resting on the septum medium.

14. A remarkable ossification of the heart, by Senac, is related; but the same is published in Senac's book. "Garanjeot opened a *Jesuit*, 72 years of age, and found in the *substance of the ventricles* (dans la substance des ventricles du cœur, a curious expression) a bone four inches and a half long, and as thick as one's thumb."

15. The following extraordinary *treatment* is by M. Fournier himself. "A soldier received a shot from a musket fired close against his breast. The ball passed directly through both lobes of the lungs, entering at the left, and passing out from the right side. Several pieces of his clothes, and of the wadding of the musket, were carried in with the ball. M. Fournier passed a *seton* through the chest, and of course through the *lungs!* By the movement of the seton every day, the foreign bodies were drawn out along with the suppuration, and in twenty-seven days the wounds were cicatrized. Several of M. Fournier's comrades remonstrated against the seton, but our author was resolute, supported by the authority of Desault, who had passed a seton on a similar occasion with success.

16. The English *Knife-eater*, who made such a noise here a few years ago, and who died at Guy's Hospital under Dr. Curry, has a conspicuous niche in the French Dictionary; but of him, "it is not our hint to speak."

17. A woman was afflicted with ascites from the age of thirty to fifty-one; during which period she was tapped one hundred and fifty-four times, ten quarts at a medium being drawn off at each operation. She never abstained from *work* during these twenty years of dropsy; and, what is not a little singular, she brought forth and suckled two fine children in that time, and was three or four times tapped during each pregnancy!

18. A child, ten years of age, by a fall on a sharp body, was wounded in the great curvature of the stomach to the extent of two inches; five stitches were taken in the wound, and in eleven weeks the recovery was perfect.

19. M. Ansiaux saw a conscript who had a hernia of the stomach, which projected through a wound in the epigastric region. The stomach could be pressed in when empty, but returned again after eating.

20. Lemery relates the case of a clergyman, who had been afflicted with periodical vomitings for eight years, accompanied with some extraordinary circumstances. For five hours before the vomiting came on, the most acute pain was felt in the kidnies. The vomitings lasted, in general, four or five hours, and consisted of a brownish watery fluid, having a *strong odour of urine* ("ayant une forte odeur d'urine.") The man ate little, but drank freely of wine. After these periodical attacks, he enjoyed good health.

21. M. Fournier was called to a man at Brussells, some time ago, who for four years had been afflicted with a most obstinate constipation of the bowels, that resisted every means of cure. On dissection, the whole of the intestinal canal was so indurated and obliterated (racornie et tellement obliterée) that a probang could scarcely be pushed through any part of it. The rectum was become nearly cartilaginous. This wretched state of the bowels was brought on by a long continued use of acetate of lead in injections, which remedy was advised by a medical man for a diarrhœa that resisted the usual means of cure.

22. In the year 1808, Professor Thomassini of Parma, saw a man, thirty years of age, of whom he relates the following curious circumstances. This man was habitually costive from his youth, but each year the torpor of his bowels increased. From the twentieth to the twenty-fourth year of his age, he had only one evacuation every eight or ten days; afterwards, at intervals of twelve days. At thirty, the date of his examination, an interval of twenty-two days took place between each fæcal evacuation. His appetite is now good, and he eats as much as two men. He becomes progressively emaciated however: his thirst is great, his urine natural in quantity and quality. No remedy nor regimen can alter the state of his bowels. Purgatives operate, but produce such debility that they cannot be persited in. His excrements are hard, dry, in the shape of small balls. Pulse frequent, heat natural.

23. M. Rivolat has communicated to the society of Marseilles a case of constipation which lasted six months, during which time there was no alvine evacuation. The patient was restored to health afterwards, and the intestinal functions reinstated.

24. M. Demet relates the case of a man, 50 years of age, who in his youth had been subject to nasal hæmorrhage, which ceased at twenty-five. From that period, he experienced incessant pains in the right side of the abdomen. At forty-three, a pain in the lumbar region was added to the former. No medicine gave any relief. He was next attacked with hæmaturia to a very alarming degree. One day, after passing much blood by urine, the patient voided per urethram a round worm fourteen inches in length, and the size of a goose quill, after which he found himself greatly relieved. The hæmaturia ceased. In the course of three months this man passed by the same channel, fifty worms of this description, but of different sizes. The patient had notice of their appearance by a sense of heat in the urinary passages, and a slight febrile movement, which went off as soon as the worms (which were dead) had been ejected.

25. Albosius relates an instance of a fœtus retained twenty-eight years in the womb. It was petrified.

26. A woman of Soigny, thirty years of age, four years married, and who had one miscarriage, became pregnant, quickened, and had a flow of fluids to the breast. At nine months, regular symptoms of labour came on; but shortly ceased. In the course of a month, she fell into a state of great debility, which continued for eighteen months, during which she was often despaired of. After this she recovered strength, but the milk continued in her breast for thirty years! She had no catamenia during this period. She died at the age of 61, of peripneumonia. A tumour, eight pounds in weight, was found attached to the fundus uteri, inclosing a male child perfectly formed, and of the natural size that might be expected at nine months pregnancy. It had four incisor teeth, two above, and two below. It did not exhibit any sign of putrefaction, nor exhale any disagreeable smell. The skin was thick, stiff, and of a yellow colour. The bones were of a larger size than those of a full grown infant. It was enveloped in a chorion and amnios, which membranes were ossified as well as the placenta. The dissection was performed in the presence of two physicians and a surgeon.

27. A fœtus was found in the abdomen of a woman, who had been pregnant twenty-three years. It had neither umbilical chord, placenta, nor membranes. It was almost entirely petrified.

28. At the end of twenty-five months of utero gestation, a woman, 45 years of age, and who had previously been the mother of eleven children, was affected with a tumor at the navel, which she opened herself, and discharged a putrid infant. She recovered. M. Fournier thinks, that in these instances (three are related somewhat similar to the last) the fœtus escaped through the parietes of the womb into the cavity of the abdomen, in consequence of abscess in the womb; and that the subsequent external abscess was the result of the irritation of a foreign body in the abdomen. The reasoning is *reasonable*.

29. The unfortunate Gilbert, a young poet, who by his eloquent satire of the "18th Century," promised to prove a second Boileau, having become insane, swallowed a key five inches and a half in length. He spoke clearly, respired easily, and complained of no pain in his throat; only had some difficulty of swallowing. From the state of his mental faculties, he was not believed when he asserted that he had swallowed the key. He frequently, however, repeated, though with an ironical smile, that the key was in his *throat*. He was taken to the Hotel-Dieu, where he was examined, but nothing extraordinary could be detected by the surgeons. Nevertheless he died. On examining the body, the key was found in the œsophagus, the ring end downwards, and the other end hooked on the aretenoid cartilages.

30. Vicq-D'Azyr relates the case of a woman, who had been subject to epileptic fits for twelve years; and which were now so frequent as four or five times a day. They always commenced with peculiar sensation in one leg, near the lower part of the gastrocnemius muscle. A medical gentleman who was present at one of these accessions plunged a scalpel into the part affected, and came in contact with a hard body, which he dissected out, and found to be a dense cartilaginous ganglion, the size of a very large pea, that pressed upon the nerve, which he divided. The woman had no return of epilepsy.

31. Vesper relates the case of an old man afflicted with hemiplegia, who presented the singular phenomenon of one half the body (the paralytic) completely jaundiced, while the other retained its natural colour. In the face it was very remarkable, as exactly half the nose was yellow and the other natural.

32. Morgagni saw a case of metastasis of urine to the brain. On opening the head of a man who died of suppression of urine, the brain was found inundated with this peculiar excretion.

33. Gastellier saw the matter of a large abscess near the malleolus externus absorbed suddenly, and translated to the stomach, from whence it was thrown off by vomiting.

34. Simorre (M. Percy relates the case) was born at Mirepoix in 1752. At the age of 15 he entered the army, and served twenty-one years in the regiment de Berry, where he arrived at the rank of captain. He had made the three campaigns of Corsica, and it was there that the young Simorre contracted the germ of his future fatal malady. He had bivouacked a long time on the marshy banks of a river, where the atmosphere was constantly obscured by vapours; when he, all at once, was seized with lancinating pains in the great toes and ankles. These had no sooner ceased, than he was afflicted with a severe ophthalmia, which, however, gave way in a short time. For several years the same succession of symptoms took place every spring, and without giving way to any medicine, terminated the same way. In course of time there was no interval of health; as soon as the ophthalmy ceased the pains commenced, and vice versa. The pains spread from the feet to the knees, and even to the hips; while his eye sight became daily weaker. In 1785, Simorre could not walk without an assistant, who also served as a guide. The year following, every joint was affected synchronously, and anchylosis made alarming progress through all the articulations. He was obliged to leave the service and retire to Metz. For a long time he bore up courageously against the ravages of disease: he felt his limbs becoming immoveable; yet, deprived of the use of several members, he braved the most exquisite sufferings in attempts at motion!

The arms and *head* (les bras et la tête) shared the same fate as the feet and knees. The whole body was rendered motionless, (le corps entier fut frappè d'immobilité.) The lower jaw (we think for *head* we should read *neck*, above) submitted to the universal immobility! Then Simorre, according to his own expression, was only a living corpse! Happy, says M. Percy, had been poor Simorre, if *insensibility* had been granted this living corpse! But far from enjoying this sad repose, Simorre, who had already suffered so much, continued still the victim of the most exquisite tortures! He lay four months on a sofa, without being able to bear the removal to a bed. The attitude which he there preserved, has evidently determined that of the skeleton, as it now appears, because the various articulations then acquired a *solidity* that rendered them ever afterwards useless. This new change occasioned the unhappy Simorre the most horrible torments. The least motion, the most gentle touch, caused him to cry out with dreadful anguish. He never slept for one moment while on this sofa of sorrow! At length he was moved into a bed; but there he spent two years of misery without ever sleeping! The moment that he attempted to close his eyes, every member was agitated with the most painful convulsions, on which opium had no effect. In 1792 his joints, which had become enormously enlarged, were quite unwieldy; and the articulating extremities of the bones were so increased in volume as to approach each other, and leave no shaft! From this time, the excessive torments, which Simorre had borne with a firmness worthy of an ancient Stoic, became much assuaged, and he could bear to be moved without experiencing much pain. He was lifted off the bed once a month, like a rigid corpse, but care was taken not to touch the *mould*, as it were, in which he lay, otherwise he suffered tortures in forming a new one. Though freed from those excruciating torments which he had formerly experienced, Simorre was still a sufferer. He never could sleep more than a quarter of an hour at a time; yet he blessed his stars that he was no worse, and amused himself with cheerful discourses and lively songs! During a great many years, he published an annual almanack of songs, composed by himself, and by the sale of which he supported himself in his state of misery! His ballads breathe the spirit of gaiety; and in them he often depicts himself in such a manner, as at the same moment to inspire mirth and commiseration!

Simorre had a good figure and a cheerful expressive countenance. His black and curled hair covered a large forehead, terminated by fine arched eyebrows. His nose was aquiline, his eyes sparkling. "This philosophical *head* (says

M. Percy,) contained the whole soul, the whole existence of Simorre!" This unfortunate officer terminated his career of earthly sufferings in 1802, aged 50 years.

Having finished this melancholy detail, on which the tear of humanity has more than once fallen during the translation, we shall here close the article "Cas Rares," which we hope has not been entirely devoid of interest or utility.

